# Intrastromal Injection of China Painting Ink in Corneas of Male Rabbits: Clinical and Histological Study

**DOI:** 10.1155/2016/8145926

**Published:** 2016-04-18

**Authors:** Alahmady Hamad Alsmman Hassan, Nesreen Gamal-Eldeen Abd Elhaliem Soliman

**Affiliations:** ^1^The Department of Ophthalmology, Sohag Faculty of Medicine, Sohag University, Sohag 82511, Egypt; ^2^The Department of Histology, Sohag Faculty of Medicine, Sohag University, Sohag 82511, Egypt

## Abstract

*Background*. Many patients with corneal opacity or complicated cataract in blind eye ask for cosmoses. In this study we tried to investigate the staining of corneas of male rabbits by Rotring China painting ink and to study the histological changes.* Method*. 10 eyes of 10 male Baladi Egyptian rabbits were injected (0.1 mL) intrastromally in the cornea by the use of China painting ink (Rotring Tinta China) through insulin syringe (27-gauge needle) by single injection; clinical follow-up is for 6 months and lastly the rabbits were scarified and the stained eyes were enucleated for histological analysis.* Results*. Clinically the stain was stable in color and distribution in corneas with no major complications. Histological results of the stained rabbit corneas showed blackish pigmentation in the corneal stroma without any inflammatory cellular infiltration. Some fibroblast cells had pigment granules in their cytoplasm in the adjacent layers.* Conclusion*. Corneal staining by China painting ink is effective and safe in staining of male rabbits cornea; however further study in human corneas with longer follow-up period is advisable.

## 1. Introduction 

Corneal tattooing started from a very old history. Many dyes and techniques are used along the history; however nowadays new advanced techniques have decreased the actual use of corneal tattooing. New techniques like corneal grafting, keratoplasty techniques, and colored contact lenses may be used for treatment of corneal opacities instead of corneal tattooing [1]. Corneal tattooing may be required in patients with corneal opacities or cataract with blind eyes with no hope in vision regain; intraocular surgery like keratoplasty and cataract surgery may be risky and expensive with no sight value with expected complications [2]. Many patients cannot tolerate tinted contact lenses. Most patients suffer much with psychological problems and persist to alter the cosmetic appearance of their eyes [3]. Corneal tattooing may be used to improve vision quality confirmed by Dr. Samuel Lewis Ziegler, in his optical indications of corneal tattooing that included albinism, iridodialysis, coloboma, aniridia, or diffused nebulae of the cornea [4]. Corneal tattooing also may be used in patients with useful vision to reduce agonising glare associated with large iris defect like surgical iridectomies or iris loss after ocular traumas [5].

Corneal tattooing in old history is firstly described by Galen of Pergamum, a Roman physician and philosopher, in 150 AD, and Aetius in 450 AD described the same technique to change the shape of disfigured eyes with leukomatous opacities [6]. Galen and Aetius in their technique cauterise the desired area of corneal surface with a heated stilet, and then they would apply the dye to the eye and they used different types of dyes such as powdered nutgalls and iron or copper sulphate [7]. This technique was used only in blind eyes with corneal leukoma with no vision [8].

In the 2nd century Galen described corneal tattooing; however the technique is not mentioned until 1869, when Louis Von Wecker described a new technique by use of black India ink to tattoo a disfiguring leukoma of the eye. In his technique he firstly used topical cocaine as anesthetic agent, then a thick black India ink solution was put over the cornea, and a multiple puncture by a grooved needle was used to insert pigment into the corneal tissue [7].

Taylor in his new technique used a bundle of needles to introduce the dye in the corneal tissue and tattooing of the eye. In 1901, Nieden used the idea of fountain pen to introduce a tattooing needle in a technique similar to the Edison electric pen. He found that this electrical needle introduces the dye more rapidly and reliably than traditional methods of tattooing. Armagnac used China ink by use of a small funnel that he fixed to the cornea and then put into the instrument and tattoo was done with a needle [8]. Morax described a new technique by splitting the corneal tissue into two vertical layers; then dye was introduced between two layers; and strong compression by dressing over the eye was done [7]. In the current study single intrastromal injection of China painting ink (Rotring) by insulin syringe in the cornea of male rabbits was done and follow-up was for six months after injection. 

*Study Design*. The study was designed as an interventional experimental study.

## 2. Material and Methods

This study was applied on 10 male rabbits weighing 1.7 to 2.8 kg with mean 2.3 kg.

**Figure 1:**
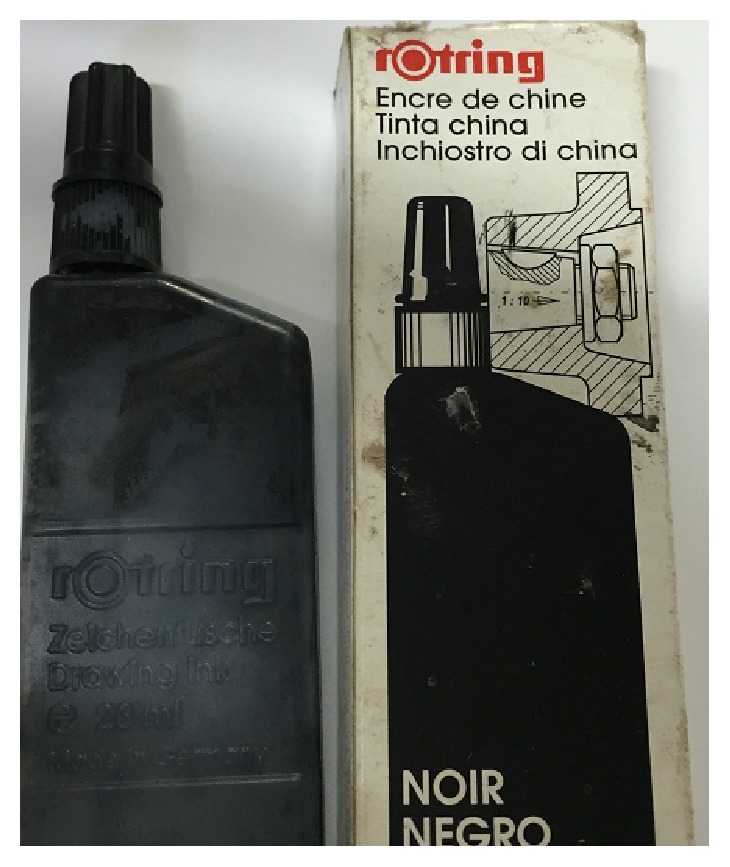


The China painting ink (Rotring) (Figure 1) sterilized in a steam autoclave for 20 minutes at 121°C in sterile glass infusion bottles.

**Figure 2:**
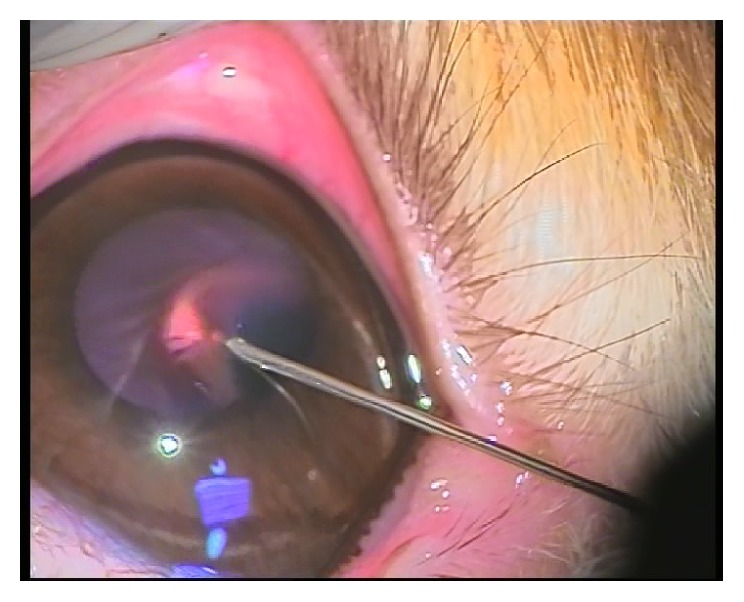


**Figure 3:**
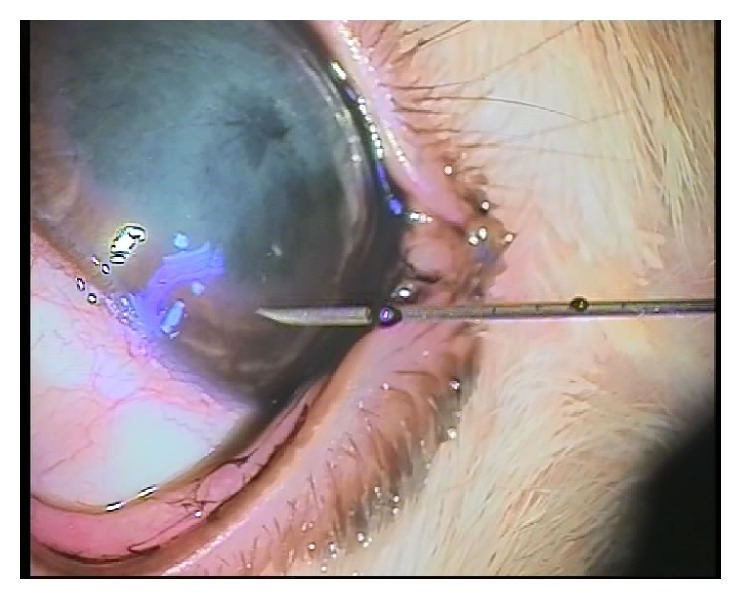


All the rabbits underwent the following technique: general anaesthesia by intraperitoneal 9 mg/kg ketamine hydrochloride. Few minutes later under the surgical microscope intrastromal injection of 0.1 mL of Rotring China painting ink by 27-gauge insulin syringe at the center of the cornea in one eye was done (Figures 2 and 3).

Clinical follow-up at the second day, third day, and every week after injection and after six-month scarification was done with enucleation of all injected eyes for further histological study. For control study we enucleated two nonstained eyes from the same animals for histological study.

### 2.1. Histological Study

Both eyes of each animal was fixed in 10% buffered formalin and processed for paraffin blocks. 5 *μ*m sections were cut and stained with hematoxylin and eosin (H&E) for routine histological study.

This study was carried out according to guidelines for animal experimentation of the Institutional Animal Care and Use committee.

## 3. Results

### 3.1. Clinical Results

**Figure 4:**
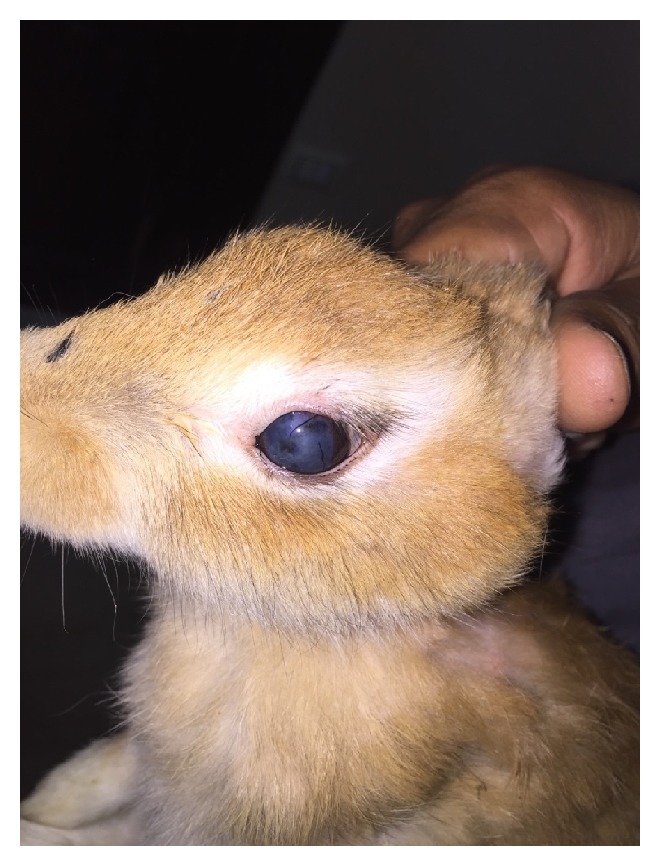
First day after injection with corneal oedema.

This study was applied on 10 male rabbits weighing 1.7 to 2.8 kg with mean 2.3 kg; at the time of injection all corneas show corneal oedema with cloudy appearance due to stromal hydration which resolved within hours leaving dark stained coloration at the center of the cornea at the site of injection (Figure 4).

All rabbits show early postoperative conjunctival redness with lacrimation which resolved early in few days after injection. In the follow-up period extended for six months no serious complications occurred with no corneal ulceration and no signs of iritis or intraocular inflammation.

In the follow-up period two rabbits died (20%), one after 3 months and the other after 4.5 months with 8 surviving until 6 months when the follow-up period finished and rabbits were scarified for corneal histological study. The death rate of rabbits is the normal death rate in Baladi rabbits in the summer season when the study was done.

**Figure 5:**
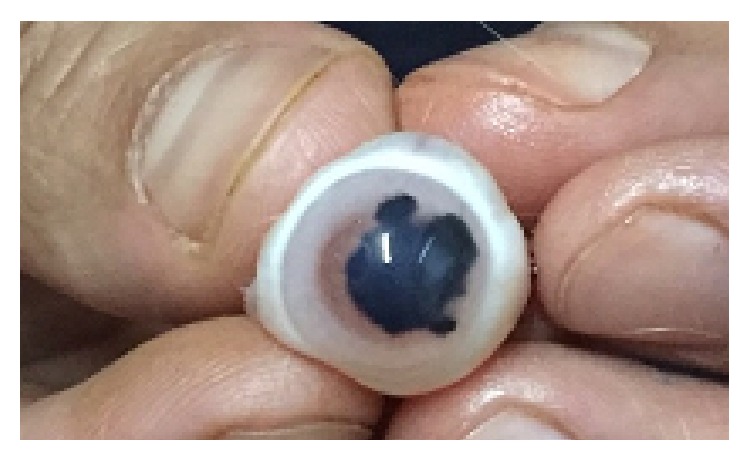
Enucleated rabbit eye with corneal staining.

During the follow-up period all injected corneas maintain the same shape and color of the blackish color of the injected ink with no fading (Figure 5).

### 3.2. Histological Results

#### 3.2.1. Group I (Control Group)

**Figure 6:**
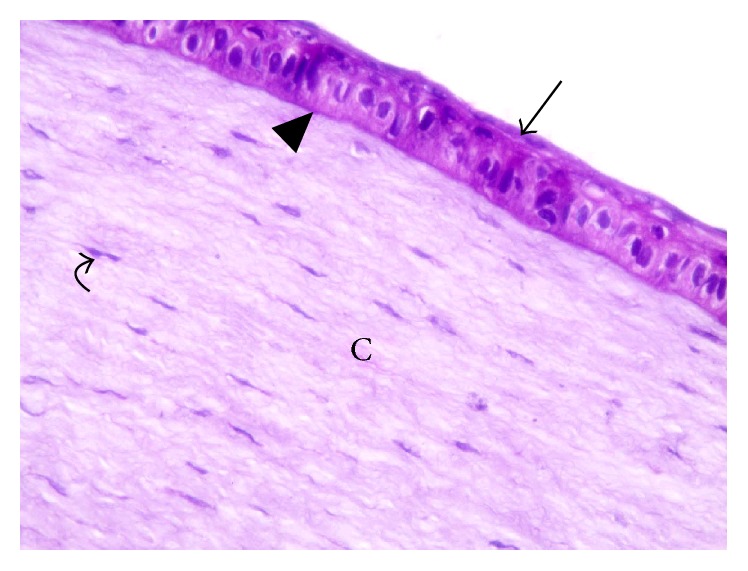
A photomicrograph of rabbit cornea group I, showing the layers of nonkeratinized squamous epithelium (arrow); basal layer, polygonal layers, and superficial layers. Notice the regular Bowman's layer (arrow head). Corneal stroma (C) and stromal cells (curved arrow). H&E ×400.

Light microscopic examination observed that the cornea consisted of five layers. The stratified squamous nonkeratinized layer appeared with its basal columnar cells, intermediate polygonal cells, and superficial squamous cells. The corneal epithelium rested on a uniform basement membrane underneath (Bowman's layer). The substantia propria was formed of regularly arranged collagen fibers and scattered spindle shaped stromal cells in between. Descemet's membrane appeared just beneath the stroma and was covered by Descemet's endothelium (Figure 6).

#### 3.2.2. Group II (Injected Cornea)

**Figure 7:**
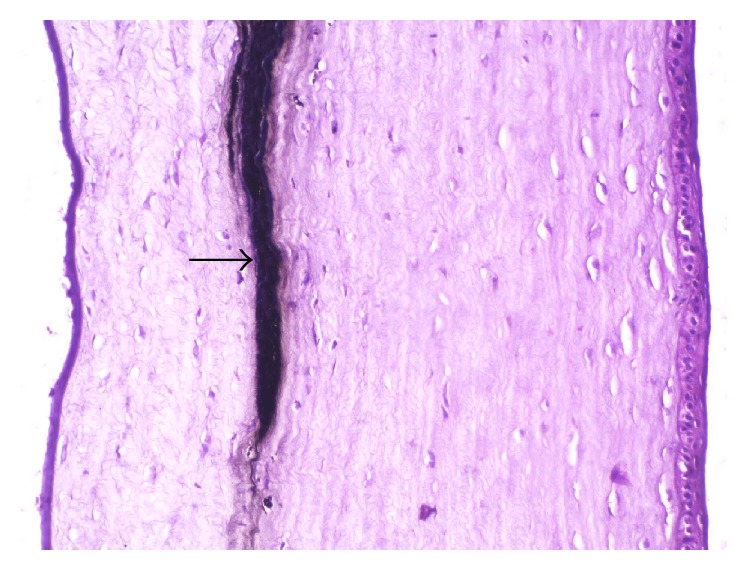
A photomicrograph of rabbit cornea group II, showing the layers of the cornea. Substantia propria contains black pigment between anterior two-thirds and posterior one-third of the cornea (arrow) with single layer of endothelial cells. Notice no infiltration of inflammatory cells in the surrounding layer. H&E ×200.

**Figure 8:**
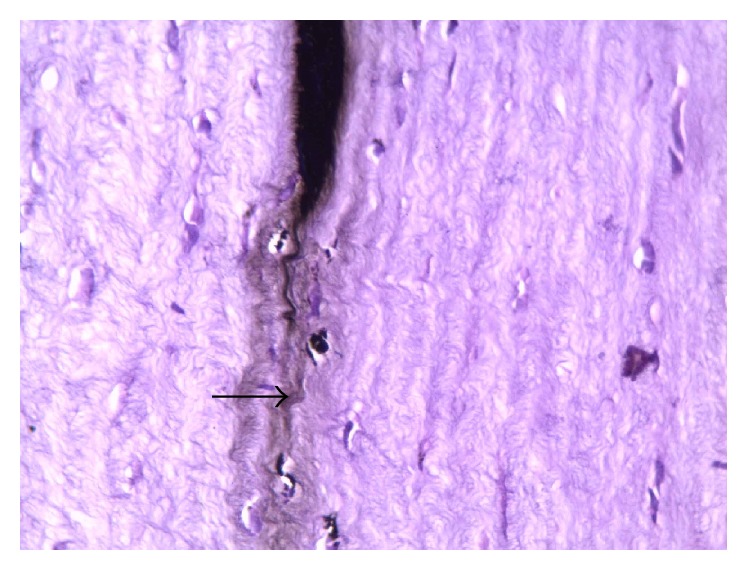
A photomicrograph of rabbit cornea group II, showing minimal diffusion of the black pigment at the adjacent corneal stroma (arrow). H&E ×400.

**Figure 9:**
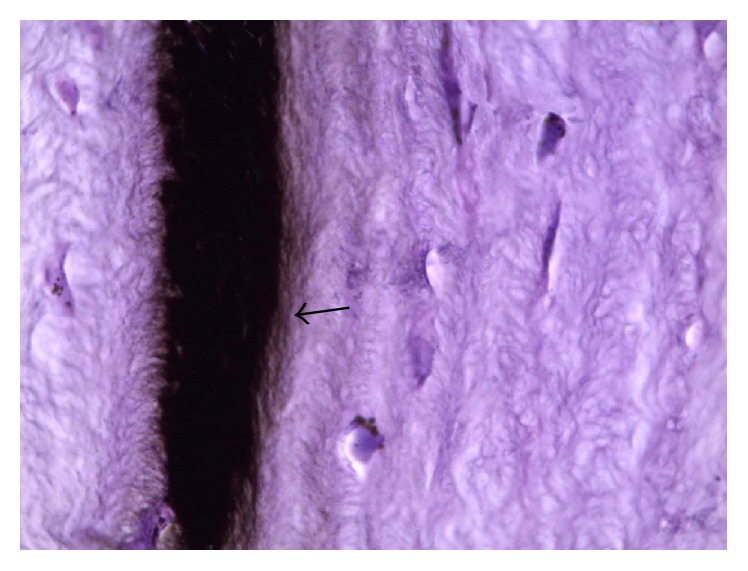
A photomicrograph of rabbit cornea group II, showing minimal diffusion of the black pigment at the adjacent corneal stroma (arrow). H&E ×400.

**Figure 10:**
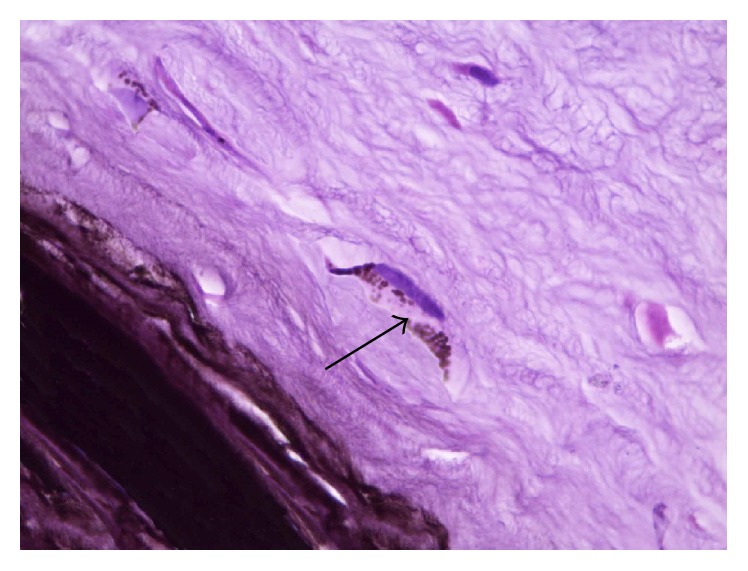
A photomicrograph of rabbit cornea group II, showing stromal cells which have pigment granules inside their cytoplasm (arrow). H&E ×1000.

Light microscopic examination of the injected cornea showed linear clumps of blackish pigment particles between anterior two-thirds and posterior one-third parallel to corneal stroma. The rest of corneal layers showed no abnormalities so they were more or less similar to control with no changes in endothelial cells (Figure 7). The pigment showed cohesiveness at all of its length. No inflammatory cellular infiltration or neovascularization was observed in the adjacent layers (Figures 8 and 9). The stromal cells appeared larger with dense nucleus. Many keratocytes near the pigmented area contain black pigment fine granules in their cytoplasm. There were no signs of fibrosis or scar formation (Figure 10).

## 4. Discussion

The corneal tattooing is an old technique that may be used in cosmetic or optical indications; the cosmetic reason is like disfiguring corneal opacity or white pupil in cases of cataract in blind eye when reconstructive surgical procedures are with no functional improvement or carry a great risk of great complications like endophthalmitis and atrophia bulbi. Tattooing may be a valuable cosmetic alternative specially with increasing difficulty in wearing a tinted cosmetic contact lens or a bulbar shell. The optical indications may be in albinism or aniridia.

Although tattooing of the cornea is an old technique with many modifications it is not widely used with difficulty to choose the optimal dye and optimal technique.

Along the history many different types of inks were used for the dyeing of the cornea. In the current study we used Rotring China painting ink which is cheap, easily available, easy in storage, and easy in sterilization as it is aqueous solution. It is available in plastic container and easy to obtain from stationary with different colors. In this study we used the black stain only. Vassileva and Hristakieva reported that ink from India is the most commonly used, providing safe and long-lasting effects [5]. Kobayashi and Sugiyama used metallic colors in powder form, various organic dyes, and uveal pigment from animal eyes. Two different methods exist: chemical dyeing with gold or platinum chloride and carbon impregnation [8].

Sekundo et al. in their comparative study between chemical and carbon impregnation reported that chemical dyeing is easier and quicker than carbon impregnation, but it fades more rapidly than nonmetallic tattooing. The most common chemical dyes that are generally used are platinum or gold chloride, which provide a jet black stain [1].

Carbon impregnation includes the use of most common inks like India ink, Chinese ink, lamp black ink, and other organic dyes [1]. Vassileva and Hristakieva, faculty members at Bulgaria universities, reported that India ink is a safe and long-lasting ink when properly diluted and it is widely used in corneal tattoo nowadays [5].

Many different techniques may be used for corneal staining. In the current study we used a single intrastromal injection of 0.1 mL of the dye with a 27-gauge insulin needle at the center of the cornea at the middle of the stroma depending on the sense of the surgeon with decreased risk of corneal ulceration or recurrent erosion. This technique is an easy noncosting and new technique. However this technique carries a major risk of accidental corneal perforation. However in the current study there are no complications from the needle during injection.

In one such method, the physician inserts the ink into the cornea stroma by multiple punctures, covering the needle with ink each time [5].

In Pitz et al. technique, they would cover a three-edged spatula needle with ink before each puncture; multiple puncture was done. Then the ink was applied into the anterior corneal stroma with each puncture [6]. Theobald would firstly inject the cornea with a needle and then rub in the ink with a Daviel curet [9].

Khan and Meyer introduced a new method of tattooing; this described technique started by removing of the corneal epithelium at the area of opacity firstly; then a piece of filter paper soaked in platinum chloride 2% would be placed by the physician onto the area for two minutes; after that a second piece of filter paper soaked in hydrazine 2% is applied for 25 seconds [10].

Thomson in his new technique in corneal tattooing of disfiguring corneal opacities used a small steel pen designed by Joseph Gillott, with the point converted into the cutting surface. The barrel of the pen would receive amount of tattooing ink for the entire operation, trying to avoid the need to refill the ink or to recover the needle with ink [11].

A new technique lamellar keratectomy with tattooing gives good results with homogeneous application of the stain [12–15].

Dermatography-like manner of tattooing has two disadvantages: firstly, the multiple puncture into the corneal stroma, enhancing activation of phagocytosis, with fading of the stain. The second disadvantage is that the multiple puncture of Bowman's layer might promote recurrent corneal erosion [6, 16].

Recently, mechanical microkeratome and femtosecond lasers have been used to create the lamellar stromal pocket for corneal tattooing [17]. Although femtosecond laser-assisted corneal tattooing demonstrates good efficacy and safety, it is very expensive [18]. In comparison to our study microkeratome and femtolaser will result in very localized and accurate size and shape of staining.

Lin et al. in their study of corneal tattooing, through anterior stromal puncture for managing painful bullous keratopathy and after follow-up period of 26 months, recurrent bullae formation occurred in 3 of 31 patients (9.68%) [19] who had undergone corneal tattooing that may match with our study in absence of inflammatory cells in histological analysis. In the current study we noticed no fading in the color of the corneal tattooing and the size and shape of pigmentation are the same all over the follow-up period. The explanation may be due to the single corneal injection decreasing the inflammatory reaction which decreases phagocytosis or vascularization responsible for fading out of the dye and this clinical result is proved by histological analysis. Six-month follow-up is considered as short follow-up period and fading of the ink may occur later on. Kim et al. in their previous long-term study on the conventional method reported that the main problem with corneal tattooing is fading color over time: 12 of 147 patients developed faded color or opaque eyes following tattooing [2]. Park et al. in their results agreed with Kim et al. in the long-term fade rate and it was similar to earlier studies on the conventional method [20].

The disadvantage of our technique of single intrastromal injection is the unpredictable size and shape of the tattooing in the stroma; however masking of different types and shapes of opacities is suggested specially if small localized lesion in the cornea is present. The limitation of our technique for blind eyes is mandatory due to unpredictable size and shape of the single intrastromal dye injection.

The histological results proved that the China ink collected in a sheath parallel to the corneal surface extracellularly between the collagen fibers. The position of the ink was between anterior two-thirds and posterior one-third. It was observed in variable thickness without leakage to the surrounding area. The pigment particles were adhesive and made a linear aggregation without diffusion in the surrounding extracellular matrix. In the present work there were pigment granules in the cytoplasm of some corneal fibroblast at the nearby storm. This might enforce the ability of the stain to be permanent and stable in the cornea as the pigment could persist in these cells for a long period. In agreement with these findings Sekundo et al. found the nonmetallic pigment particles in the keratocyte in the corneal stroma but not the metallic ones in patient cornea [1]. Fujita et al. 1987 reported the presence of the ink pigment after tattooing both intracellularly and extracellularly. The ability of fibroblast to acquire a phagocytic activity to ingest the pigment as an attempt to protect the cornea from damage by foreign nontoxic materials was established [21].

In a study by Kobayashi and Sugiyama a 64-year-old woman who underwent keratopigmentation for corneal opacities in both eyes 53 years ago was examined by confocal microscope; the examination showed scattered highly reflective particles in a geographic pattern were observed in the superficial stroma near Bowman layer. In addition, clusters of highly reflective granules were in the midstroma to superficial stroma [8]. The absence of the inflammatory cells and neovascularization could support the safety of the stain that we choose for the process of tattooing. Moreover, the stromal cells secrete anti-inflammatory cytokines to prevent the reaction which affect the clarity of the corneal tissue [22].

## 5. Conclusion

Intrastromal injection of Rotring China painting ink in corneas of rabbits is safe, easy, simple, and effective technique and the histological results in the present study gave sufficient evidence that pigment is safe to use in patient for the future work. However six-month follow-up is a short period to evaluate the possible toxicity or fading of the dye. Further study in human corneas in indicated patients is advised with longer follow-up period.
